# Radiation-free extraction of intrahepatic biliary ascariasis in a pregnant patient using peroral cholangioscopy: a case report

**DOI:** 10.1055/a-2608-0545

**Published:** 2025-07-02

**Authors:** Yufan Ma, Yongfeng Yan, Ji Zheng, Qin Zhang

**Affiliations:** 1 159413 Department of Gastroenterology, The First Peopleʼs Hospital of Liangshan Yi Autonomous Prefecture, Xichang, Sichuan Province, China

A 23-year-old woman at 7 weeks of gestation was admitted with a 2-day history of abdominal pain. Abdominal ultrasonography identified biliary ascariasis ( Fig. 1 ), while gynecological ultrasound confirmed a viable intrauterine early pregnancy. Laboratory findings were unremarkable. In light of the patient’ s desire to continue the pregnancy, a radiation-free endoscopic approach for worm extraction via oral cholangioscopy was planned.

**Fig. 1:**
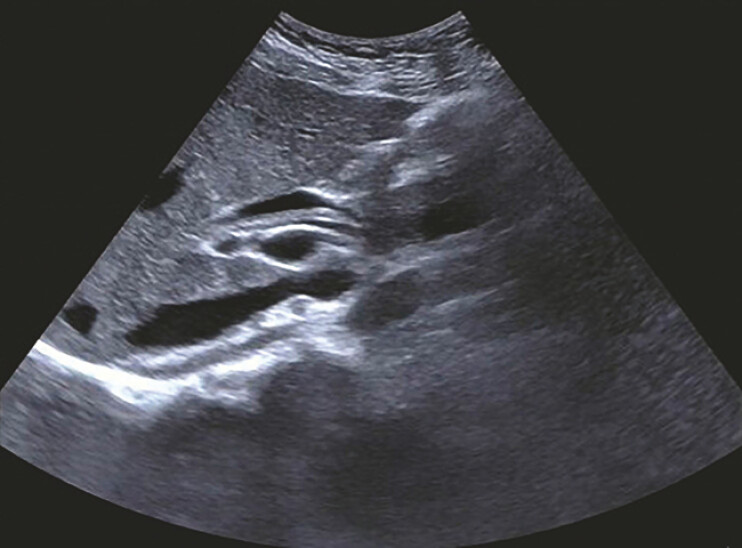
Abdominal color Doppler ultrasonography identified dual parallel linear hyperechoic structures within the biliary tract, exhibiting no acoustic shadowing.

Cannulation was successfully performed using a wire-guided sphincterotome. Aspiration of yellow bile confirmed the guidewire’s entry into the common bile duct ( Fig. 2 ). The sphincterotome was withdrawn smoothly after bowing. Given that the migration of the Ascaris into the bile duct had sufficiently dilated the papilla, sphincterotomy was deemed unnecessary and omitted. The EyeMax (Micro-tech) was advanced into the common bile duct along the guidewire, and the ascaris was successfully extracted ( Video 1 , Fig. 3 ). The patient experienced no postoperative complications.

**Fig. 2:**
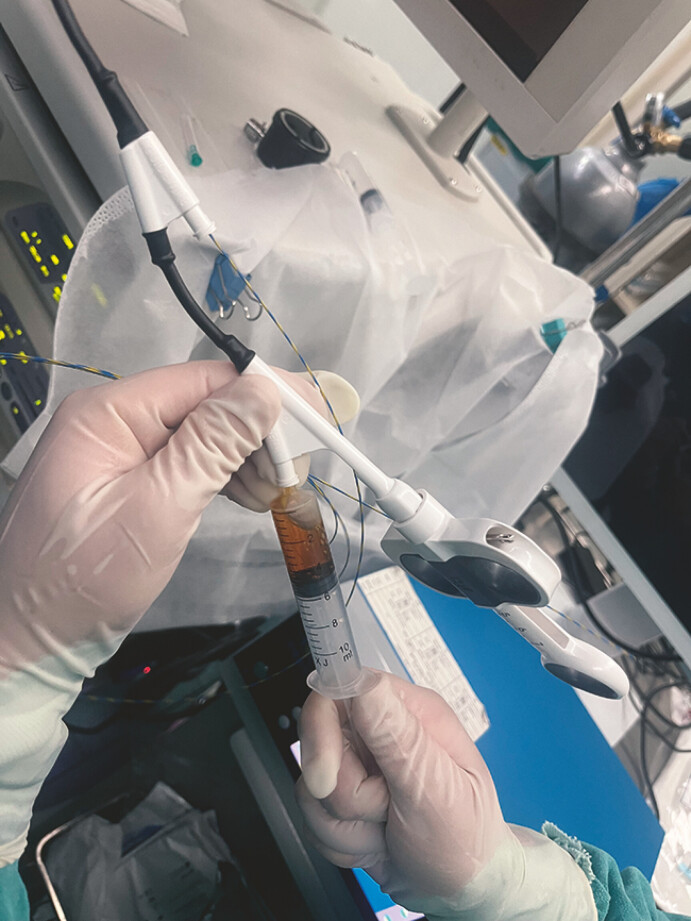
Following the advancement of the sphincterotome into the lumen over the guidewire, aspiration of yellow bile was achieved, confirming successful cannulation of the bile duct.

Radiation-free extraction of intrahepatic biliary ascariasis via peroral cholangioscopy.Video 1

**Fig. 3:**
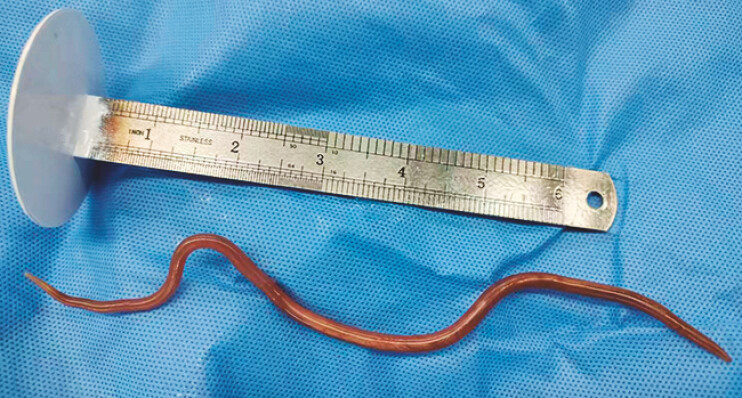
The externally retrieved Ascaris lumbricoides.

Pregnancy is a well-documented risk factor for biliary ascariasis 1 . While endoscopic retrograde cholangiopancreatography (ERCP) remains a critical therapeutic modality, its associated radiation exposure poses potential risks to the fetus. In this context, oral cholangioscopy offers a safe and efficient alternative for ascaris removal without radiation exposure. Notably, a novel endoscopic retrograde direct cholangioscopy (ERDC) technique has been developed to facilitate visible biliary duct cannulation 2 , further enhancing the feasibility and safety of this approach.

Endoscopy_UCTN_Code_CCL_1AZ_2AI

Endoscopy_UCTN_Code_CCL_1AC_2AG

## Notes

Endoscopy E-Videos https://eref.thieme.de/e-videos
*E-Videos* is an open access online section of the journal *Endoscopy* , reporting on interesting cases and new techniques in gastroenterological endoscopy. All papers include a high-quality video and are published with a Creative Commons CC-BY license. Endoscopy E-Videos qualify for HINARI discounts and waivers and eligibility is automatically checked during the submission process. We grant 100% waivers to articles whose corresponding authors are based in Group A countries and 50% waivers to those who are based in Group B countries as classified by Research4Life (see: https://www.research4life.org/access/eligibility/ ). This section has its own submission website at https://mc.manuscriptcentral.com/e-videos .
